# Potentiation of GPR68 alleviates post-ischemia BBB dysfunction and brain edema in mice

**DOI:** 10.1016/j.expneurol.2026.115850

**Published:** 2026-05-22

**Authors:** Wenyan Sun, Wenqi Fu, Youfang Zhou, Virendra Tiwari, Xiang-ming Zha

**Affiliations:** Clinical Neuroscience Research Center, Department of Neurosurgery, Tulane University School of Medicine, New Orleans, LA 70112, United States of America

**Keywords:** GPR68, Hemorrhagic transformation, Brain edema, Blood-brain barrier (BBB), Protein extravasation, Neuroprotection

## Abstract

Ischemia-reperfusion can increase blood-brain barrier (BBB) permeability and extravasation of peripheral molecules. Further damage to BBB results in blood vessel rupture and hemorrhagic transformation (HT). Associated with these processes, brain edema is common. In previous studies, we and others have shown that Ogerin, a small molecule positive modulator of the acid-sensitive GPR68-reduces ischemic brain injury. Here, we asked whether Ogerin attenuates post-ischemia BBB leakage, HT, or edema. To determine HT and edema, we first analyzed the 2,3,5-triphenyltetrazolium chloride (TTC) images from a previous study. In male mice, Ogerin administration reduced both HT severity and brain edema. Ogerin had no significant effect in GPR68−/− mice. Ipsilateral brain tissue exhibited increased mouse IgG and reduced ZO-1 and NeuN, and Ogerin showed a trend of increasing Claudin-5 clustering at cerebral microvessels. In female animals, Ogerin significantly reduced post-stroke edema while showed a trend of reducing HT severity. Combining TTC staining and a sensitive and quantitative fluorescent approach to detect Evans Blue, we examined BBB permeability and brain injury at 72 h. Ogerin reduced brain infarct, edema, and Evans Blue extravasation on day 3 after reperfusion. These results suggest that GPR68 activation may protect against post-tMCAO BBB hyperpermeability, alleviate HT, and reduce brain edema. These findings, together with the earlier observations on brain injury, suggest that pharmacological potentiation of GPR68 is a promising therapeutic intervention for improving post-ischemia outcomes.

## 1. Introduction

Stroke is one major cause of long-term disability with significant morbidity and mortality, causing substantial health burdens (Banzrai et al., 2023; Ren et al., 2025). Ischemic stroke, which constitutes about 85% of all stroke cases, results from the occlusion of a cerebral blood vessel and subsequent loss of cerebral blood flow (Flynn et al., 2008). Reperfusion therapies, including intravenous tissue plasminogen activator (tPA) and endovascular thrombectomy, restore blood flow and improve stroke outcome. During ischemia and following reperfusion, metabolic disruption and inflammation lead to endothelial injury, leading to dysfunction of the blood-brain barrier (BBB) (Jiang et al., 2018; Krueger et al., 2015; Krueger et al., 2017; Yao, 2019). The initial dysfunction includes increased permeability and protein extravasation. Further progression of the injury results in rupture of blood vessel and post-stroke hemorrhagic transformation (HT) (Bustamante et al., 2016; Garcia-Yebenes et al., 2018; Hong et al., 2021; Yang et al., 2019). Clinically, depending on patient characteristics and treatment parameters, HT occurs in approximately 3–40% of patients with ischemic stroke (Chen et al., 2024; Spronk et al., 2021). In parallel with post-stroke BBB dysfunction, brain edema occurs. This is a result of cytotoxicity as well as increased vascular permeability (Khatri et al., 2012; Lin et al., 1993; Rosenberg and Yang, 2007; Yang and Betz, 1994). Elevated edema can propagate injury into peri-infarct tissue and exacerbate HT. Both HT and edema worsen neurological outcomes after stroke. These findings highlight the need for strategies that preserve cerebrovascular integrity following ischemia-reperfusion.

During ischemia, and following reperfusion, the disruption of metabolism and elevated inflammation lead to prolonged brain acidosis (Farkas and Rose, 2025; Zha et al., 2022). Acidosis in the brain activates various proton-sensitive receptors. G protein-coupled receptor 68 (GPR68, also known as OGR1), a proton-sensitive GPCR, exhibits predominant neuronal expression (Wang et al., 2020b; Xu et al., 2020). One study reported the presence of GPR68 in a fraction of endothelial cells in small diameter artery (Xu et al., 2018). In brain ischemia, we and others have demonstrated that GPR68 activation leads to neuroprotection and reduces ischemic brain injury (Li et al., 2024; Sun et al., 2024; Wang et al., 2020b). Pharmacological potentiation of GPR68 using a positive allosteric modulator Ogerin (Huang et al., 2015) reduces infarct volume and improves neurological outcomes in mouse tMCAO (Li et al., 2024; Sun et al., 2024). Genetic deletion of GPR68 is associated with an upward trend in HT severity at both early and delayed time points following ischemic stroke (Wang et al., 2020a). This result raises the question of whether Ogerin protects BBB or reduces edema after ischemia. Addressing this question can offer new insights into the potential impact of GPR68 for long-term ischemia outcome.

In this study, we first analyzed triphenyltetrazolium chloride (TTC)-stained brain sections from our previous study to determine HT and edema, and performed correlation analysis between brain infarct size and HT level. With Western blotting, we asked whether Ogerin alters post-stroke mouse IgG extravasation, ZO-1 or NeuN expression. We then asked whether the Ogerin has similar effect in female mice after tMCAO. Lastly, we examined the effect at ~72 h after reperfusion to answer whether Ogerin alters delayed injury and BBB dysfunction.

## 2. Material and methods

### 2.1. Mice

Breeding colonies of wild-type (WT; GPR68+/+) C57BL/6 and GPR68-knockout (GPR68−/−) mice on a congenic C57BL/6 background. Knockout lines were periodically backcrossed to WT C57BL/6Ncrl mice every 5–10 generations. WT C57BL/6 mice were either generated from in-house breeding or purchased from Charles River. Male mice aged 9–15 weeks and female mice aged 13–18 weeks of age were used for previous dataset (see (Sun et al., 2024)). The animals for surgery, biochemical, and histological analysis in this study were 3–7 month old, both males and females. All animals were housed under standard laboratory conditions. All procedures adhered to the National Institutes of Health Guide for the Care and Use of Laboratory Animals and were approved by the Tulane University Institutional Animal Care and Use Committee.

### 2.2. Material and reagents

Ogerin was purchased from Sigma (SML1482), Cayman (#34228), and Tocris (#5722). Antibody used including: Rabbit anti-tubulin (1:20 k for WB, Proteintech, #80713–1-RR), mouse anti-tubulin (1:20 k for WB, University of Iowa Developmental Hybridoma Bank, E7), Rabbit Neun (1:2 k for WB, CST #12943), and Rabbit ZO1 (1:1 k for WB, Invitrogen TJ275967); rat anti-CD31 (1:500 for IF, BD Pharmingen, MEC13.3, #553370); mouse anti-claudin 5 (1:500 for IF, Invitrogen, #35–2500). Secondary antibodies used for WB include donkey anti rabbit IRdye 800 (1:10 K, (Li-Cor Biosciences, #926–32,213) and donkey anti-mouse IRdye 680 (1:10 K, Li-Cor Biosciences, #926–68,022); for IF, Alexa or dylight 488-conjugated donkey-anti-mouse, Alexa or Dylight 555-conjugated donkey anti-rabbit, Alexa 649 conjugated donkey anti-rat were purchased from Invitrogen or Jackson immunological, and all were used at 1:1 k.

### 2.3. Transient middle cerebral artery occlusion (tMCAO)

Transient focal ischemia was induced using the Longa model as described previously (Longa et al., 1989; Sun et al., 2024). Male (27 ± 4 g) and female (23 ± 3 g) mice were randomly assigned to experimental groups. All surgeries and scoring were conducted under blinded conditions. Mice were anesthetized with 1.5–2% isoflurane in a 70% N_2_O/30% O_2_ mixture, and body temperature was maintained at 37 ± 0.5 °C using a feedback-controlled heating pad. Cerebral blood flow (CBF) was continuously monitored via laser Doppler flowmetry (MoorVMS-LDF2) with the probe fixed to the skull using cyanoacrylate adhesive. A silicone-coated monofilament (Doccol Corp.) was inserted into the external carotid artery (ECA) and advanced into the internal carotid artery (ICA) to occlude the middle cerebral artery (MCA). We used either #702023 (for body weight less than 28 g) or #702223 (for body weight 28–34 g) suture in this study. After 60 min of occlusion, the filament was withdrawn to allow reperfusion. For CBF analysis, mean flow values were recorded before occlusion, during occlusion, and after reperfusion (average of a 1–2 min peak value). Mice were included for analysis only if CBF was reduced to 5–20% of baseline during occlusion and recovered to ≥55% upon reperfusion. All flow traces were reviewed independently by at least two investigators to ensure consistency. For 24 h HT and edema analysis, we re-analyzed the TTC images from our previous study (Sun et al., 2024). For 72 h outcome, and for Western blot and immunostaining experiment, we performed new surgeries in this study.

### 2.4. Vaginal cytology

For female mice, we performed vaginal cytology to determine the stages in estrus cycle (Sun et al., 2024). Approximately 50 μl of distilled water was used to gently flush the vaginal canal with a 200 μl pipette tip. The collected fluid was spread onto glass slide, air-dried, and stored at −80 °C. Slides were later stained with 2% crystal violet for 30 s, rinsed, and examined by light microscopy to determine the estrous stage based on cellular morphology.

### 2.5. Intravenous administration of Ogerin

At reperfusion, we delivered Ogerin via retro-orbital intravenous (i.v.) injection under isoflurane (Sun et al., 2024). We dissolved Ogerin in DMSO to 20 mM, and aliquoted Vehicle (DMSO) or Ogerin into 20 μl aliquots and blind-coded the tubes. On the day of injection, we added 200 μl of saline per 20 μl aliquot and used this working solution during the same day. For injections at reperfusion, we injected slowly into the retrobulbar sinus via the inferior conjunctival fornix using a 27–30 G needle at ~45° to minimize tissue damage and ensure complete delivery. For repeated doses on day 1 and 2, we delivered Ogerin via tail vein. Based on our previous study which showed effective dose at 1 mg/kg while more effective at 2–2.5 mg/kg (Sun et al., 2024), we used the 2 mg/kg dose for the rest of experiments.

### 2.6. Brain infarct, Evans Blue extravasation, and brain edema ratio

The 24 h cohort brain infarct values for the along with its quantification were presented in our previous study (Sun et al., 2024). For edema, we calculated the % ratio as:

Edema(%)=100×(ipsilateralvolume-contralateralvolume)/contralateralvolume.

For the 24-h brain injury, as detailed in the previous study (Sun et al., 2024), mice were euthanized 24 h after 60 min tMCAO, and brains were coronally sectioned into 1 mm slices. Sections were incubated in 2% 2,3,5-triphenyltetrazolium chloride (TTC) solution and subsequently fixed in 4% paraformaldehyde.

To analyze Evans Blue extravasation and brain injury on day 3. We performed tMCAO for 45 min, delivered Ogerin at reperfusion (retroorbital), day 1 & 2 (through tail vein), at 2 mg/kg for each injection. On day 3 post-tMCAO surgery, we injected intravenously Evans Blue (EB) dye (2%), 100 μl per 25 g body weight. After 2 h of circulation, we perfused the mice with saline, isolated the brain, and sectioned into 2 mm thickness, and performed TTC staining. We chose this protocol based on previous studies which showed the feasibility of obtaining both injury and extravasation data from the same brain (Huang et al., 1998; Kuts et al., 2019). Following TTC staining, we scanned the brain sections with a scanner to capture TTC staining pattern and imaged the same sections with a Keyence X1000 fluorescent microscope. For fluorescent imaging of EB in slices, we used a 1.25× NA 0.04 and a Cy5 filter because EB shows excitation peak at 620 nm and emission peak at 680 nm (Saria and Lundberg, 1983). To gain more quantitative measurement, we then collect the left and right brain separately, weight the brain tissue, and add 5× volume of saline relative to the tissue weight. After homogenizing the tissue, we added TCA to 50% and incubated at 4 °C for 1–2 days to allow efficient extraction of EB from the tissue. We centrifuged the mixture at 15,000*g* for 20 min, transferred 80 μl of the supernatants into a 96-well plate. For calibration, we prepared the standard EB solutions of various concentrations (0, 0.01, 0.03, 0.1, 0.3, 1, 10, 30, 100 μg/ml) in the saline: TCA buffer, and added 80 μl of the standards into the 96-well plate. We then added 120 μl of ethanol per well and measured the fluorescence at 620 nm excitation and 680 nm emission using a fluorescent plate reader.

### 2.7. Evaluation of hemorrhagic transformation (HT)

To determine HT severity on TTC stained sections, we adopted a method as described by an early study (Garcia-Yebenes et al., 2011). Similar to our previous study (Wang et al., 2020a), we assigned the HT categories in this study as following: 0: No detectable hemorrhage. I-III: spotty-type (or petechial) with 1–3, 4–10, or >10 microhemorrhages, respectively. IV-V: Parenchymal hemorrhage which contains at least one hemorrhagic area beyond the petechial type. For IV, the HT area is less than 10% of the ipsilateral side in all sections; For V, at least one slice shows HT area ≥10% of the ipsilateral side of the brain section.

#### Brain isolation, lysate preparation, and Western blotting.

Brain tissue isolation and Western blotting analysis were performed similarly as described in earlier studies (Sun et al., 2024; Zhou et al., 2021). Briefly, at the designed time point, we perfused the mice with ice cold phosphate buffered saline (PBS) or saline and rapidly isolated the brains. Next, we quickly dissected the tissue between +1 and −2 AP position, separated into ipsilateral and contralateral areas, and lysed the tissue in lysis buffer (PBS with 1% Triton X-100, 0.4% SDS, and freshly added protease inhibitors and phosphatase inhibitors). We sonicated the lysates, centrifuged, and quantified with protein concentration using a Rc-Dc protein assay kit (Bio Rad). We then adjusted protein concentration and added one-half volume of 3× SDS sample buffer (with 15% β-mercaptoethanol). Prior to loading, we incubated the samples at 45C for 20 min. For gel electrophoresis, we ran 10% SDS-PAGE to separate the proteins of interest. Following transferring to nitrocellulose membranes, we blocked the membranes in blocking buffer (0.1% casein in 0.2 × PBS pH 7.1–7.5) for 30–60 min, added primary antibodies in blocking buffer containing 0.1% Tween-20, and incubated at 4C overnight with gentle rocking. Following washing and secondary antibody incubation at room temperature for 1 h, we imaged the membranes with a GelDoc imager (Bio-Rad) according to manufacturer’s instructions. We used ImageJ to perform densitometry analysis of image bands. Primary antibodies used in the study include Rabbit tubulin (Proteintech) 1:20 K, mouse tubulin (University of Iowa Developmental Hybridoma Bank) 1:20 K, rabbit NeuN (Cell Signaling Tech) 1:2 K, and ZO1 (Invitrogen TJ275967) 1:1 K; Secondary antibodies used in the study include Alexa or Dylight 680- or 800-conjugated donkey anti-rabbit and donkey anti-mouse antibodies (1:10 K).

#### Immunostaining and fluorescent imaging.

For immunofluorescence, we followed similar protocols as described in our previous studies (Sun et al., 2024; Zhou et al., 2021). Briefly, we perfused animals with 2% paraformaldehyde, post-fixed overnight in 1% paraformaldehyde, protected through 30% sucrose, performed cryosection at 20 μm. Following permeabilization with 0.3–0.5% Triton X-100 for 30 min, we performed regular immunostaining using the CD31 (1:500) and Claudin-5 (1:500) antibodies and donkey-anti-rat Alexa 649 and donkey-anti-mouse Alexa 488 antibodies. For imaging, we used a Keyence X-1000 fluorescent microscope, with Plan Apo N 1.25× NA 0.04, Plan Apo 20× NA 0.75, or Plan Apo 60× NA 1.2 objectives. For analysis, we used ImageJ and Adobe Photoshop.

#### Dataset and Statistical analysis.

For 24 h HT and edema analysis, we re-analyzed the TTC images collected in our previous study (Sun et al., 2024). For Western blot, immunostaining, and 72 h BBB permeability and injury, we performed new surgery and analysis. We performed statistical analyses using GraphPad Prism. We used χ^2^ test for trend to compare HT grade distributions. For quantification of hemorrhagic area and edema values, we first analyzed sample normality and then performed either 2-tailed *t*-test or Mann-Whitney *U* test. For relationship between edema and infarct size, we performed linear regression analyses with an F test. For multiple-tests, we performed Holm-Sidak correction. The number of animals used are included in the Figures. We plotted bar graphs using mean ± SEM. Statistical significance was defined as *p* < 0.05 unless otherwise noted; ns = not significant.

## 3. Results

### 3.1. Ogerin attenuates hemorrhagic transformation and reduces edema after tMCAO

**Fig. 1.**
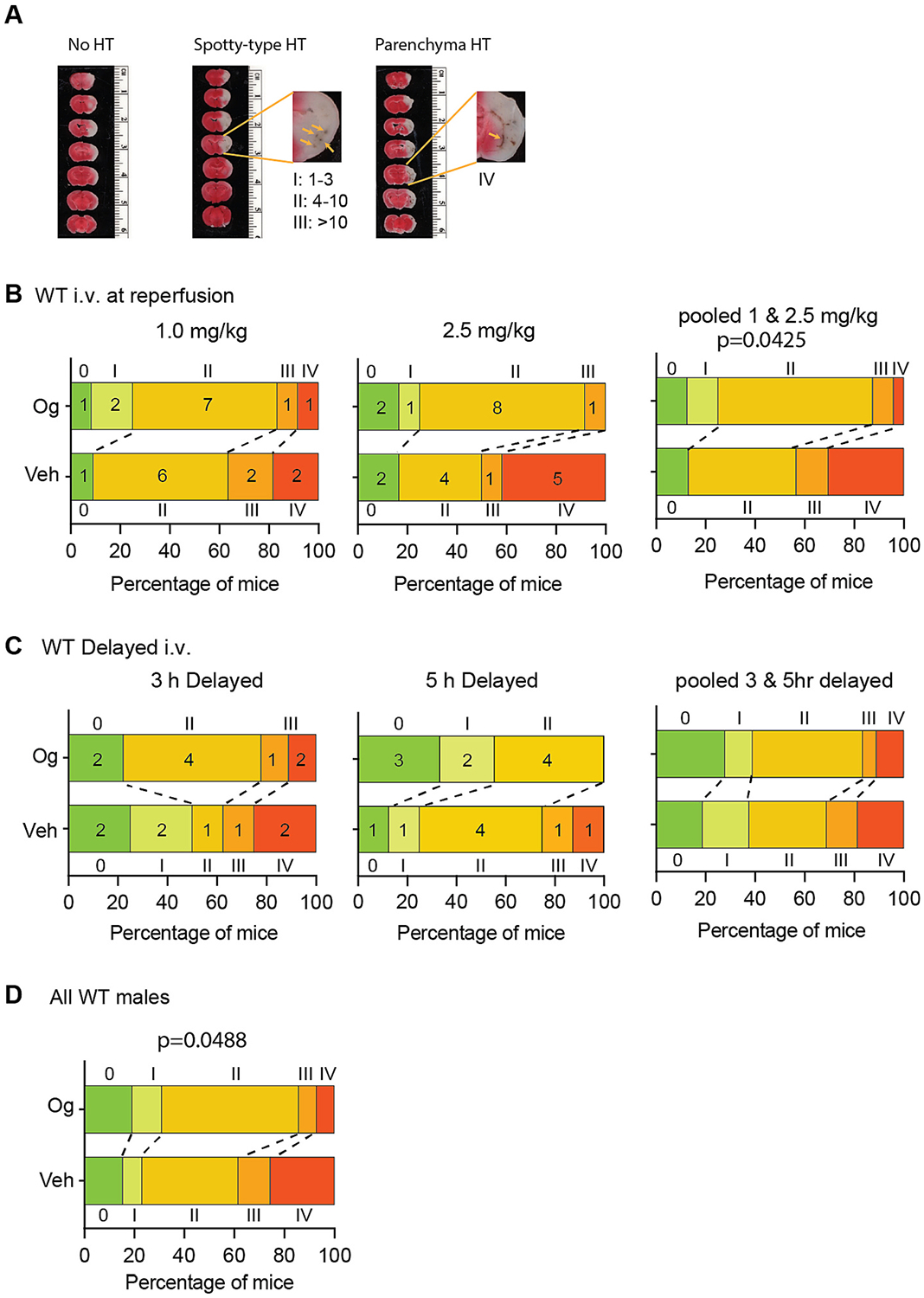
Ogerin reduces hemorrhagic transformation (HT) at 24 h after tMCAO. (A) Representative images and schematic illustrating HT categories: 0, no HT; I–III, spotty-type HT with 1–3, 4–10, or > 10 microhemorrhages respectively; IV, parenchymal HT. (B) Distribution of HT grades in WT male mice subjected to tMCAO and treated with vehicle or Ogerin at 1.0 or 2.5 mg/kg. (C) Effect of delayed Ogerin delivery following reperfusion in WT male mice. Following tMCAO, vehicle or Ogerin (2.0 mg/kg, i.v.) were administered at 3 h or 5 h after reperfusion. (D) Distribution and quantification of combined HT categories in pooled WT tMCAO male mice. *p* value was from χ^2^ test for trend. Numbers on the bars show the number of different animals in each category, each from a distinct surgery. For the pooled graph in B & C, the numbers are the summation of the individual categories on the left. For D, the numbers are the summation of all animals from B & C.

To answer whether enhancing GPR68 function protects against HT after tMCAO, we re-analyzed TTC-stained brain sections from our previous study (Sun et al., 2024). In this analysis, we adopted a similar method described in earlier studies (Garcia-Yebenes et al., 2011; Wang et al., 2020a). We scored the severity of HT as the following: category 0, no HT; categories I-III, spotty/petechial type HT with 1–3, 4–10, or > 10 microhemorrhages respectively; and category IV, larger area HT beyond petechial type (Fig. 1A).

We first examined the effect of Ogerin administered at reperfusion. In vehicle-treated group, about half of vehicle-treated mice exhibited less than 10 microhemorrhages while one-third of the mice had >10 microbleeds or contained larger area hemorrhages (Fig. 1B). Ogerin i.v. at either 1.0 or 2.5 mg/kg led to a trend of reduced proportion of HTs in category III and IV. The difference was not significant for either dose alone. Given that these groups showed similar trends, we adopted a previous approach which pools different datasets from randomized controlled trials to increase statistical power (Bangdiwala et al., 2016). Though with the caveat that the cohorts have differences, pooling can be a valid approach for exploratory studies for efficacy. For this reason, we pooled the groups and analyzed them together here and at several other places in this study (see below). The pooled analysis showed a significant improvement with Ogerin treatment (Fig. 1B, right graph, χ^2^ test for trend, *p* = 0.0425). We next assessed the effect of Ogerin (2.0 mg/kg) administrated at delayed time points. At either 3 h or 5 h after reperfusion, the Ogerin-treated group exhibited a trend of reduction in the HT-III & IV categories (Fig. 1C). the Ogerin group showed a significant reduction in HT severity as compared to the vehicle group (χ^2^ test for trend, *p* = 0.0482).

**Fig. 2.**
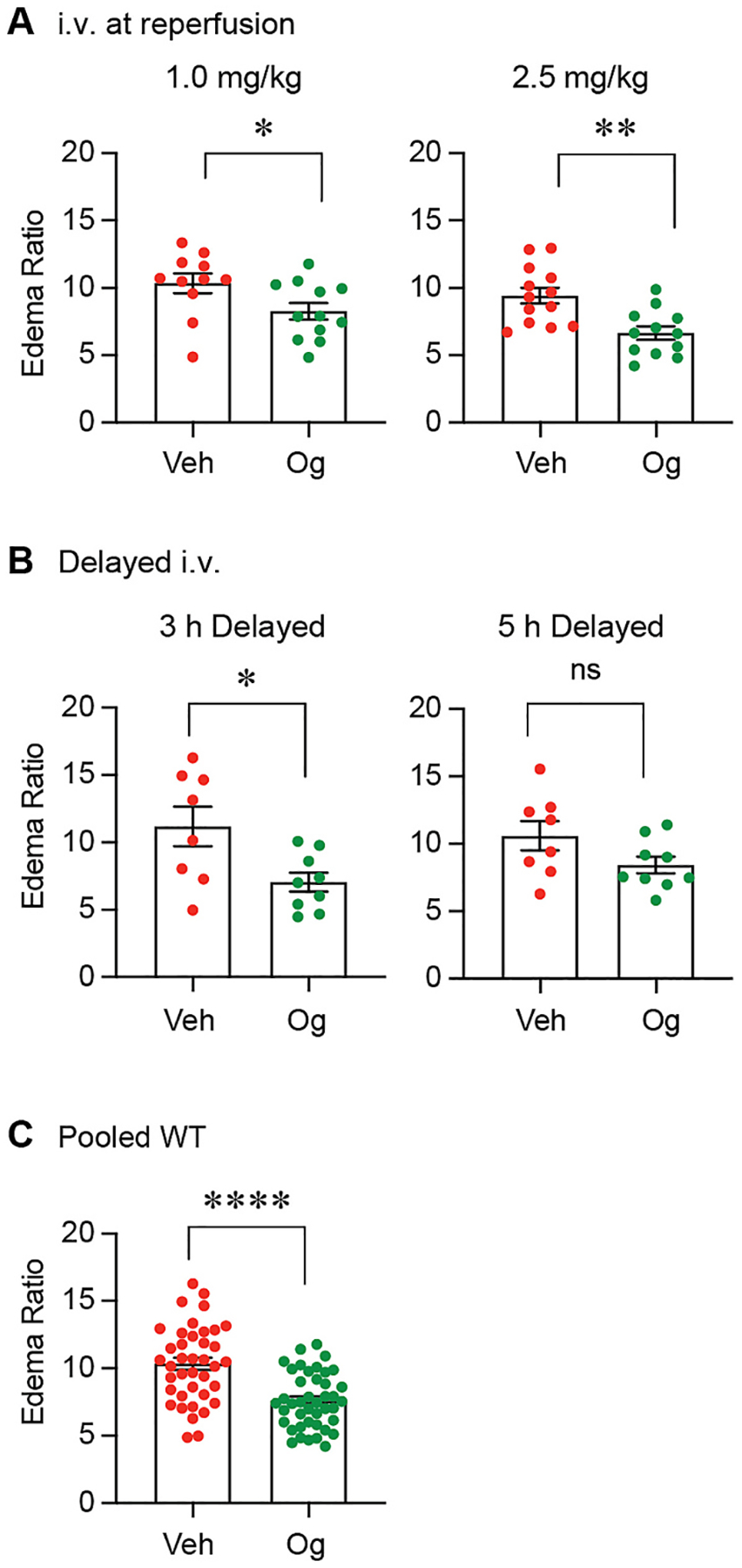
Ogerin treatment reduces post-tMCAO edema at 24 h in male mice. (A) Brain edema ratio in WT male mice subjected to tMCAO and treated with vehicle or Ogerin at 1.0 or 2.5 mg/kg. (B) Brain edema ratio in WT tMCAO male mice treated with Ogerin administered 3 h or 5 h after reperfusion. (C) Quantification of edema ratio of all male mice pooled together. * *p* < 0.05 ** *p* < 0.01 **** *p* < 0.0001; two-tailed *t*-test. The animals here are the same as in Fig. 1.

Next, we quantified contralateral and ipsilateral brain areas and calculated % edema. At both 1 and 2.5 mg/kg dose, Ogerin delivered at reperfusion reduced post-MCAO edema (Fig. 2A). Delayed IV at 3 h also reduced edema (Fig. 2B). We observed a similar trend for delayed delivery at 5 h. When pooled all groups together, the vehicle and Ogerin group exhibited an average edema rate of 10.34% ± 2.84% vs 7.59% ± 2.04% (*p* < 0.0001, 2-tailed *t*-test), respectively (Fig. 2C).

**Fig. 3.**
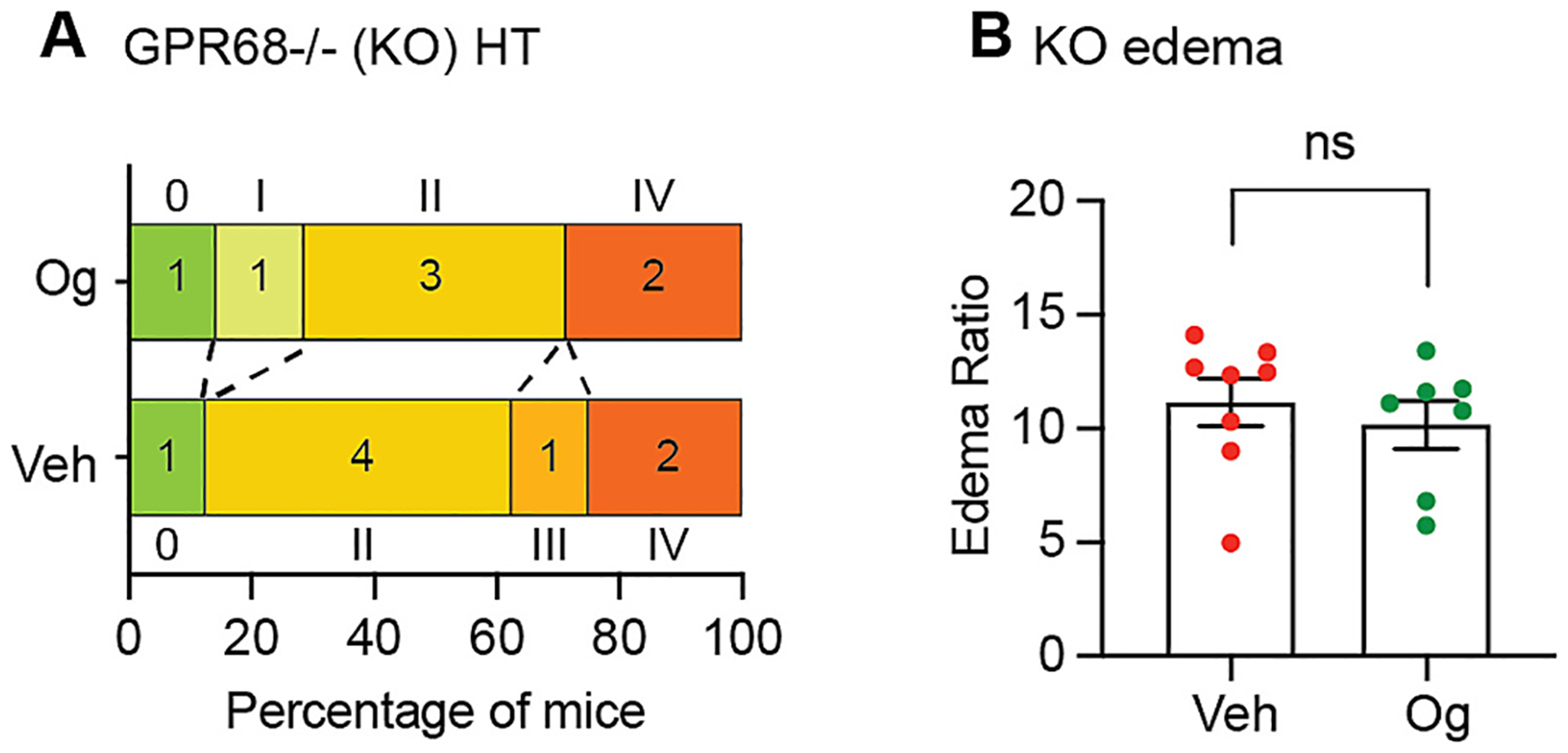
Ogerin does not alter post-ischemia HT or edema in GPR68−/− mice. (A) Distribution and quantification of HT severity scores in GPR68−/− (KO) mice. (B) Brain edema ratio in KO mice treated with vehicle or Ogerin. Summary bar graphs show mean ± SEM. The number of animals is shown on the bars, with each one from a separate surgery.

To determine whether the protective effect of Ogerin depends on GPR68, we analyzed vehicle and Ogerin-treated GPR68−/−. The two groups showed comparable severity of HT and similar levels of edema (Fig. 3). These findings indicate that Ogerin attenuates post-ischemia HT and edema in a GPR68-dependent manner.

### 3.2. Ogerin reduces infarct size and edema in male tMCAO mice with moderate HT

**Fig. 4.**
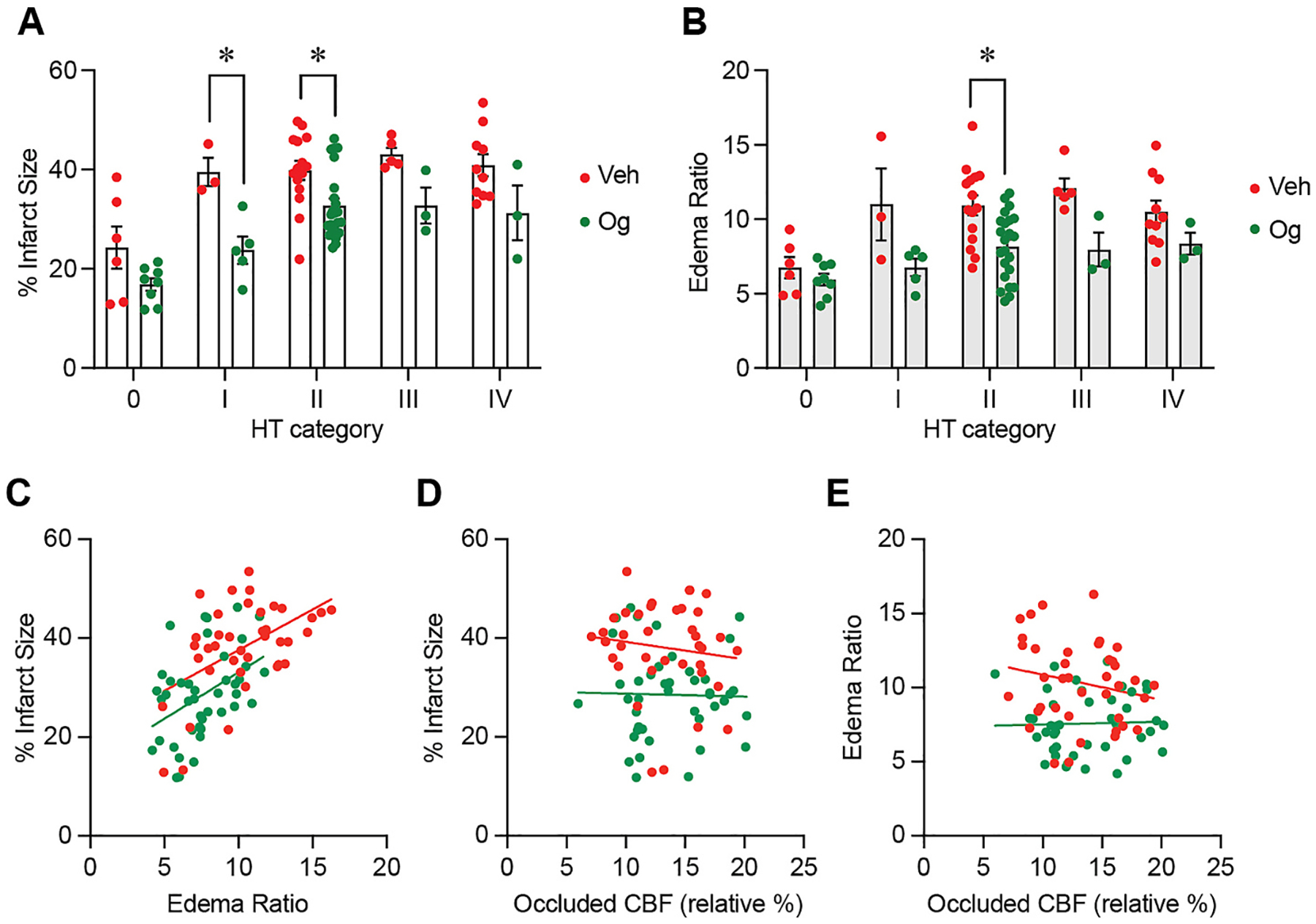
Ogerin reduces % infarct at moderate HT and the effect is independent on CBF during the occlusion phase. (A) Infarct size by HT category in male tMCAO mice treated with vehicle (red) or ogerin (green). For analysis in Fig. 4, all male WT mice, including the cohorts of IV at reperfusion, 3 h, and 5 h, were pooled together to increase the N for comparison. (B) Edema ratio by HT category. (C-E) Linear regression analysis showing the relationship between % infarct- edema ratio (C), % infarct- occluded CBF (D), and edema ratio- occluded CBF (E). The animals are the same cohort as in Figs. 1 & 2, with each dot representing a separate animal.

Our previous study showed that Ogerin reduced infarct percentage after tMCAO. To gain more insights into the effect of Ogerin, we examined the correlation between injury, HT, and edema. For this analysis, we pooled all male mice to increase the statistical power. In the vehicle group, HT-0 group showed an average infarct size of 24.3 ± 4.3% (Fig. 4A). In contrast, the average infarct from HT-I to HT-IV was similar at around 40%. Ogerin-treated group showed a similar trend of reduced infarct in all HT categories, with that in HT-I & -II to be significant (Fig. 4A, multiple t-test with Holm-Sidak correction). For edema vs HT category analysis, we observed a similar pattern. In vehicle group, % edema was 6.8% at HT-0 and increased to ~11% at HT-I through HT-IV (Fig. 4B). Ogerin treatment did not affect edema in the HT-0 group but all other HT categories showed the same trend of reduced edema. These data suggest that Ogerin reduces brain infarct and edema across different severities of HT, although the effect becomes relatively smaller at HT 0 or IV categories.

Next, we examined the correlation between infarct size and edema. The scatter plot showed that vehicle and Ogerin groups exhibited a similar slope (F test, slope, *p* = 0.2435) but the Ogerin group downshifted for about 5% (F test, intercept, p < 0.0001) in the infarct-edema regression line (Fig. 4C). In our surgery, we monitored cerebral blood flow for the entire duration of MCAO surgery. As stated in methods, our inclusion criteria for analysis is a consistent blocked blood flow between 5 and 20% of the starting value. We next plotted the infarct and edema over the % CBF blocked. Either the injury or the edema does not show apparent changes at different values of blocked CBF (Fig. 4D, E). However, compared to the vehicle group, Ogerin-treated mice showed a downshift in both % infarct and % edema.

GPR68 potentiation with Ogerin has no major impact on post-tMCAO extravasation of mouse IgG.

**Fig. 5.**
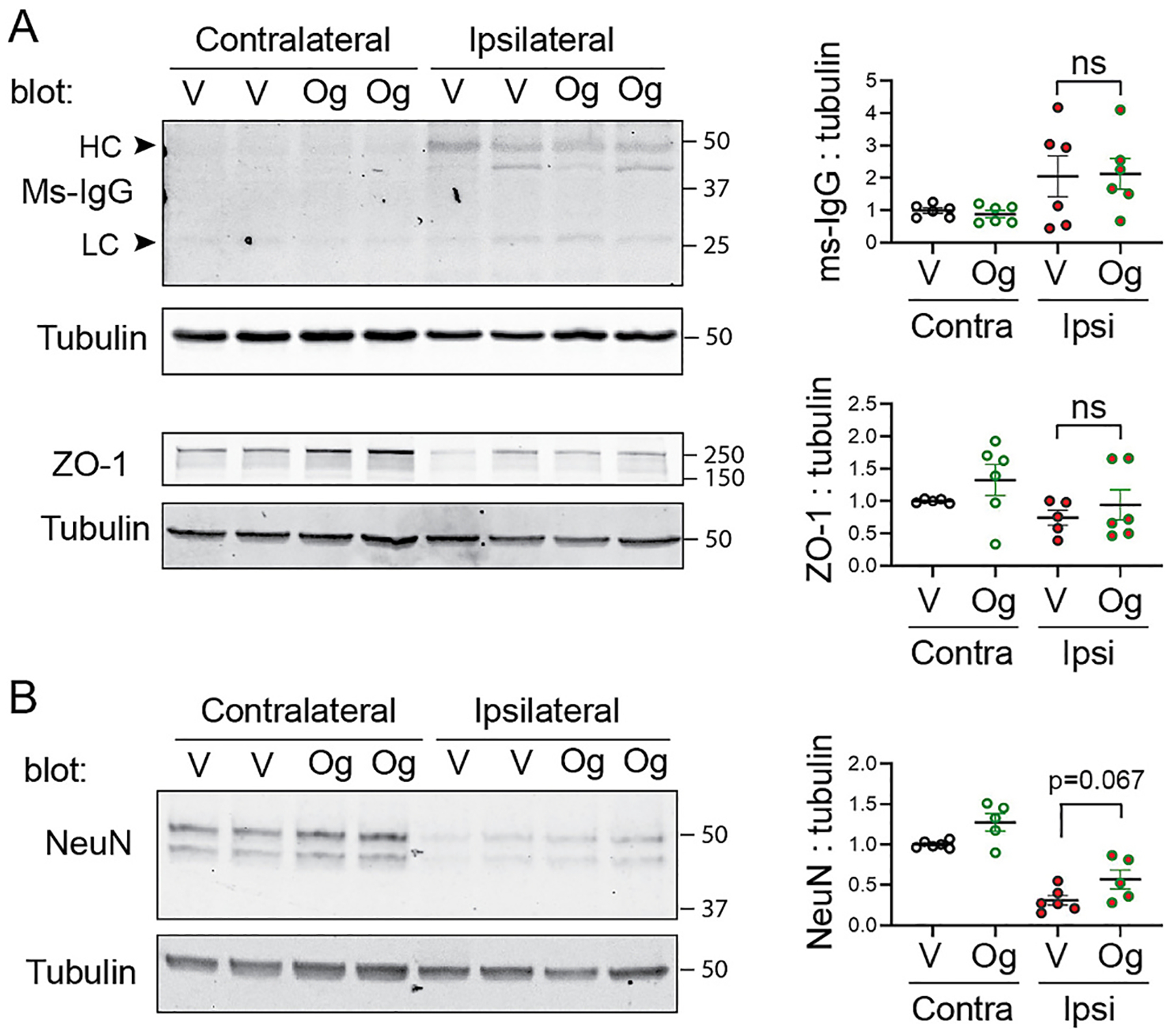
Ogerin does not block post-tMCAO IgG extravasation. Representative Western blot and summary bar graph for post-tMCAO levels of mouse IgG (A), ZO-1 (B), and NeuN (C). Tissues were isolated 24 h after tMCAO. Each dot represents one animal, *N* = 5–6.

Extravasation of plasma proteins occurs after ischemia-reperfusion. To determine whether Ogerin reduces post-ischemia BBB hyperpermeability, we performed 60 min tMCAO and intravenous delivery (i.v.) of vehicle or Ogerin at reperfusion. At 24 h after reperfusion, we perfused the animals with saline, isolated ipsilateral and contralateral brain tissue, and performed Western blotting analysis. To detect the leakage of plasma proteins, we blotted for mouse IgG. In sham control animals or contralateral brain tissue, there were little signal of mouse IgG (Fig. 5A). In contrast, we detected increased level of mouse IgG in ipsilateral brain tissue following tMCAO. Ogerin-treated group exhibit similar amount of mouse IgG in the ipsilateral brain. ZO-1 exhibited similar reduction in vehicle- and Ogerin-treated ipsilateral brain tissue (Fig. 5A). In contrast, Ogerin showed a trend (*p* = 0.067, 2 tailed *t*-test) of attenuating tMCAO-induced reduction of NeuN (Fig. 5B).

**Fig. 6.**
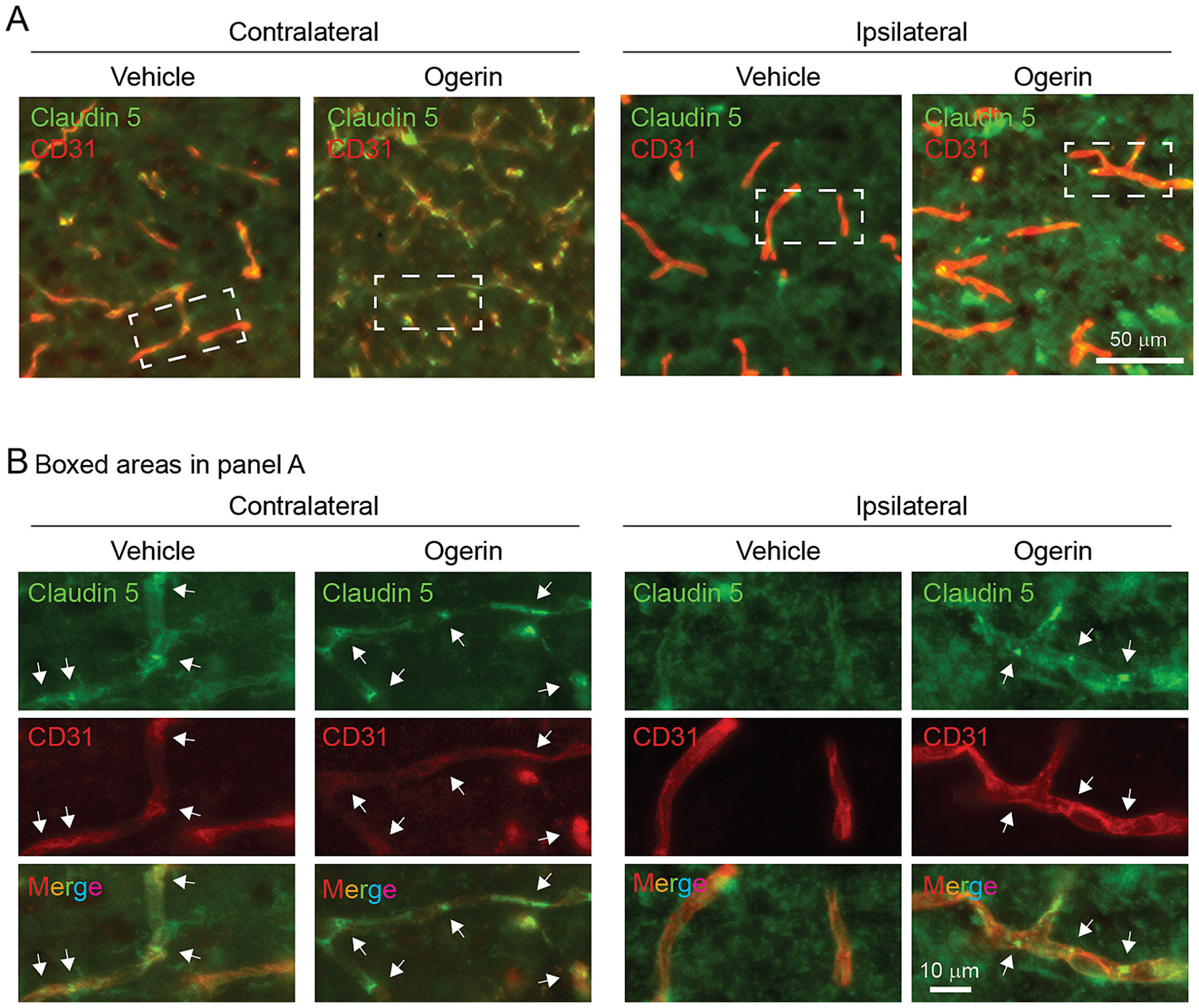
Ogerin on post-ischemia clustering of Claudin-5. Representative images showing the immunofluorescence of Claudin-5 (in green) and CD31 (pseudo colored in red) in somatosensory cortex at 24 h after tMCAO. (A) Lower magnification view. (B) Higher magnification view of the boxed areas in panel A. Arrows in B point to clusters of claudin puncta. Note that, when compared with the vehicle group, the Ogerin-treated group tends to have increased puncta in both contralateral and ipsilateral tissue.

To visualize the changes in endothelial cell junction, we performed immunostaining. At 24 h after reperfusion, we perfuse-fixed the animal, performed cryosection, and stained with CD31 and Claudin-5 antibodies. In contralateral cortex in both vehicle- and Ogerin-groups exhibited comparable patterns of staining, although there might exist a trend of increased punctate pattern of Claudin-5 in the Ogerin group (Fig. 6A, B). Ipsilateral cortex (Fig. 6) and striatum (not shown) lost most of the vessel-associated clustering of Claudin-5. With Ogerin treatment, we observed a trend of increased Claudin-5 puncta in some microvessels (Fig. 6B, right most panel).

### 3.3. Ogerin exhibits similar protective effect in female mice

**Fig. 7.**
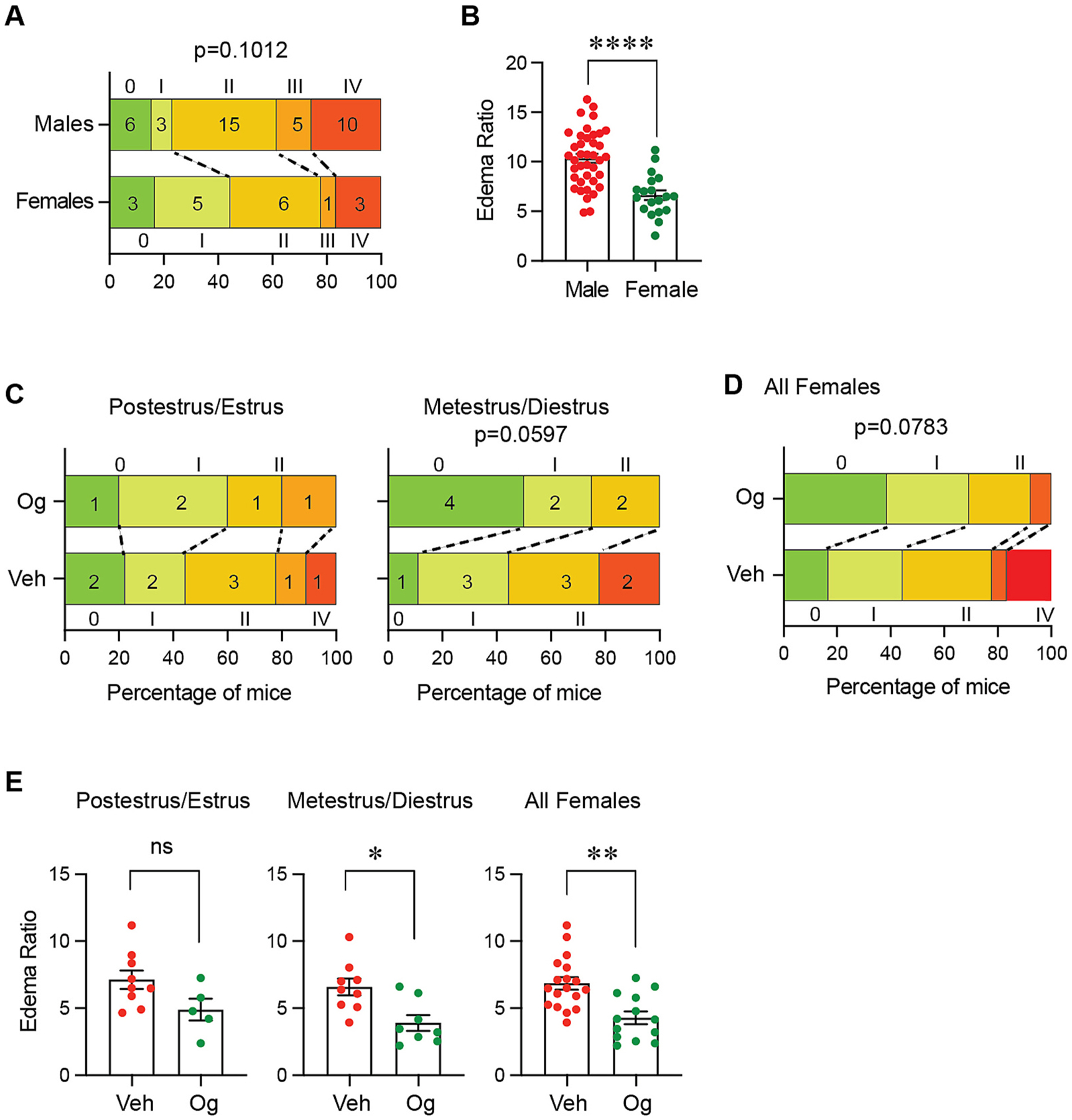
Ogerin reduces post-stroke hemorrhagic transformation and edema in female mice. (A, B) Comparison of HT (A) and edema (B) between male and female vehicle treated mice. (C) Distribution of HT categories in proestrus/estrus (left) and metestrus and diestrus (right) stages in female mice following tMCAO. (D) Distribution and quantification of hemorrhagic transformation categories in pooled female mice. (E) Brain edema ratio in proestrus/estrus (ns), metestrus/diestrus (*P* < 0.05), and pooled female mice. * p < 0.05; ** p < 0.01 two-tailed student’s t-test. Data are presented as mean ± SEM. The numbers on bars are individual animals. The male in panel A is the same vehicle cohort in Fig. 1D. For panel C & E, the vehicle group is the same cohort as panel A female, but separated according to stages in the estrus cycle as indicated.

The above analysis focus on male animals. To determine whether Ogerin has similar effect on HT or edema in female, we analyzed TTC images obtained from female mice. We first compared the male vs female vehicle-treated groups. As expected, the females exhibit a trend of lower HT and significant reduction of edema as compared to the males (Fig. 7A, B). As stated in our previous study, we determined their estrus stage for female animals with vaginal cytology (Sun et al., 2024). We combined the mice in proestrus and estrus stages which show relative higher estrogen level and those in metestrus and diestrus stages when progesterone level is higher. Ogerin treatment had little effect on HT in proestrus/estrus stage but showed a solid trend (*p* = 0.0597) of reducing HT in diestrus/metestrus stages (Fig. 7C). The combined analysis of all females maintained a trend of reduced HT in Ogerin-treated group (Fig. 7D). Lastly, we analyzed edema in female mice. Ogerin treatment led to a trend toward reduced edema in proestrus/estrus group (Fig. 7E). For metestrus/diestrus stages, or when all females are pooled together, Ogerin significantly reduced edema ratio. These findings imply a potential cerebrovascular protective role for GPR68 activation in both sexes after ischemic stroke.

### 3.4. Ogerin reduces BBB leakage and brain infarct on day 3 after tMCAO

**Fig. 8.**
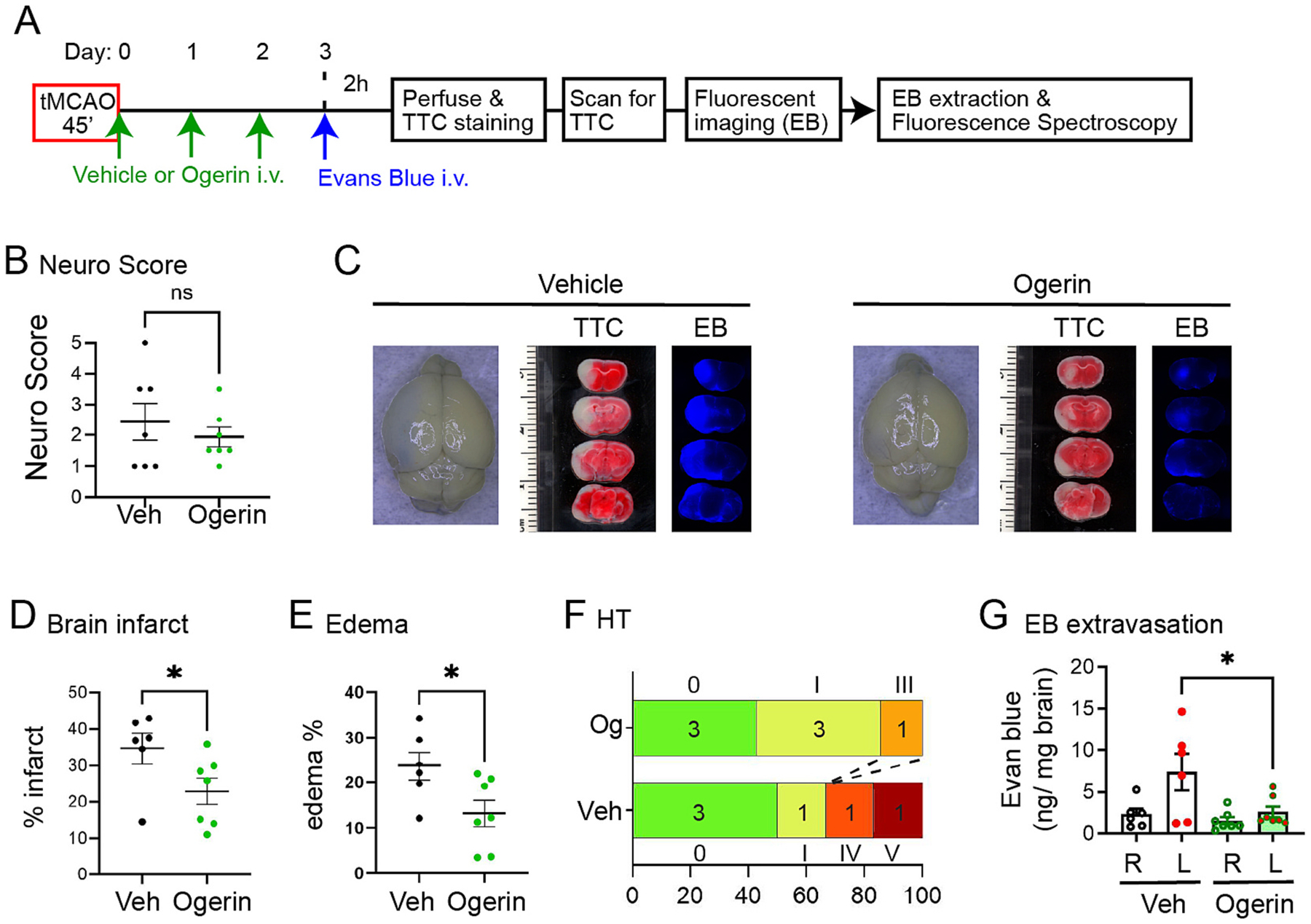
The protective effect of Ogerin at delayed (day 3) time point. (A) Diagram showing the timeline of the day 3 experiment. (B) Summary diagram for neurological scores. (C) Representative set of images showing the whole brain view, TTC staining, and Evans Blue fluorescence (Ex620/Em 680 nm). (D-G) Quantification showing brain infarct (D), brain edema (E), HT (F), and Evans Blue extravasation (G). * p < 0.05, Mann-Whitney *U* test (panel D, non-normal distributions). two-tailed student’s t-test (panel E), or one-way ANOVA (panel F). R- contralateral side; L- ipsilateral side. Each dot represents one separate mouse.

Following ischemia, HT and edema continue to evolve beyond 24 h (Jickling et al., 2014). To examine the outcome at a delayed phase, we performed tMCAO for 45 min on day 0, daily IV delivery of Ogerin for 3 days (day 0, 1, 2). On day 3, we assessed neurological performance, performed IV of Evans Blue, perfused the mice with saline 2 h later, and performed TTC staining (see timeline in Fig. 8A). There were no differences in neurological scores between the two groups (Fig. 8B). Following scanning for TTC stained sections, we imaged the same set of slices with fluorescent microscopy using the Cy5 filter (Fig. 8C). The infarct size for vehicle was 34.7 ± 4.2% (Fig. 8D). The Ogerin-treated group exhibited an average infarct of 22.9 ± 3.6%, significantly smaller than that of the vehicle group (*p* = 0.0350, Mann-Whitney test). In parallel with the reduced infarct size, Ogerin treatment reduced brain edema (Fig. 8E). There were no significant changes in HT categories (Fig. 8F). However, the mice which showed parenchymal type HT were 2 in 6 for the vehicle group and 0 in 7 for the Ogerin group. To determine EB extravasation, we first quantified the fluorescence intensity of EB on the fluorescent images. In this analysis, we noticed that the area of EB fluorescence did not completely overlap with TTC-revealed infarct area. The Ogerin-treated group showed a solid trend (*p* = 0.0541, ANOVA with Tukey post hoc) of reduced EB fluorescence in the ipsilateral (L) tissue when compared to the vehicle group. To gain more quantitative measurement of EB level in brain tissue, we extracted EB, performed fluorescent optometry, and calibrated the fluorescence against a series of EB standards. The ipsilateral brain in the vehicle group showed EB level of 7.4 ± 2.2 ng per mg tissue (Fig. 8G). In the Ogerin group, the ipsilateral brain tissue showed EB level of 2.6 ± 0.7 ng/mg tissue, significantly lower as compared to the vehicle-ipsilateral side (*p* = 0.0308, Anova with Tukey post hoc test).

## 4. Discussion

Our data here supports several conclusions. First, Ogerin reduces post-tMCAO HT and alleviates brain edema. Second, these protective effects depend on GPR68. Third, Ogerin treatment does not prevent post-tMCAO extravasation of immunoglobin. Fourth, Ogerin-induced protection at BBB likely extends to females. Lastly, Ogerin IV reduced ischemic injury, brain edema, and EB extravasation at 3 days after reperfusion. This result is consistent with the previous report of protective effect of Ogerin at 24 h, but suggests that Ogerin leads to protection at delayed time points. These findings bring a possible role regarding Ogerin and GPR68 in cerebrovascular protection. Previous studies have reported protective effect of Ogerin against ischemic neuronal injury (Li et al., 2024; Sun et al., 2024). Together, these findings suggest that potentiation of GPR68 with Ogerin improves post-stroke outcome through reducing neuronal injury and alleviating the development of post-stroke HT and edema at both early (6–24 h) and delayed (~72 h) phases after reperfusion.

Following transient ischemia, the BBB exhibits different degrees of permeability, ranging from no leakage, protein extravasation but with intact endothelial cell morphology, to basement membrane disruption and HT (Krueger et al., 2015). In clinic, early HT occurs in a significant fraction of stroke cases and is a major determinant of post-stroke mortality and poor functional recovery after ischemic stroke (Chen et al., 2024; He et al., 2022; Paciaroni et al., 2008). The current result shows that activating GPR68 does not stop the extravasation of mouse IgG. Instead, Ogerin treatment reduces post-ischemia HT. This finding suggests that GPR68 activation offers protection against ischemia-induced vascular rupture. This speculation is consistent with our day 3 result which showed 2 out of 6 mice in the vehicle group showed large HT as compared to the 0 out of 7 mice in the ogerin group. Our result on day 3 further shows that Ogerin-induced protection extended to at least 72 h after reperfusion, and resulted in lower brain infarct and edema. One limitation of this study is that the lower N at the 72 h experiment does not allow us to draw a statistically significant conclusion on HT. However, the reduced Evans Blue extravasation supports a protective effect on BBB. One technical note on this experiment is that detecting EB fluorescence provides a sensitive measurement for BBB leakage and also enables us to obtain quantitative measurement of both brain injury and BBB extravasation from the same animal.

We do not know the exact mechanism for GPR68-dependent protection at BBB. GPR68 is widely present in brain neurons while GPR68 activation or potentiation of its function reduces ischemia-induced brain infarct (Li et al., 2024; Sun et al., 2024; Wang et al., 2020b; Xu et al., 2020). In the correlation analysis of infarct size vs HT category, Ogerin treatment reduced infarct percentage in animals which exhibited spotty-type HT. This result suggests a possibility that, through reducing ischemic neuronal injury and injury-associated inflammatory responses, Ogerin slows the transformation of BBB leakage to rupture. Nevertheless, one study reported the presence of GPR68 in a fraction of endothelial cells in small diameter artery (Xu et al., 2018). Possiblity exists in that part of the protective effect of GPR68 at BBB is through a direct effect on this small cohort of GPR68-expressing endothelial cells.

Increased brain edema contributes to poor functional outcomes and increased mortality (Battey et al., 2014; Simard et al., 2024). As one measurement of edema formation, we used the relative areas of ipsilateral vs contralateral side to quantify edema. This method is not an accurate measurement of brain edema but allows us to draw a qualitative conclusion. Our result here demonstrated that Ogerin reduces post-tMCAO brain edema. This finding is consistent with a neuroprotective effect for GPR68 activation as described earlier (Li et al., 2024; Sun et al., 2024; Wang et al., 2020b). After reperfusion, the edema formed has a mixed contribution from multiple mechanisms, including cytotoxic ion disturbance, increased BBB leakage, and impaired drainage (Gerriets et al., 2009; Gotoh et al., 1985; Krueger et al., 2015; Loubinoux et al., 1997). While edema associates with injury and HT, the development of edema and HT can diverge in a time-dependent manner after ischemia (Hamann et al., 1996; Jin et al., 2010). In this study, the 5 h delayed drug delivery led to reduction of edema but did not have a significant impact on HT. Future studies are warranted to investigate the exact mechanism for how GPR68 regulates post-ischemia edema and BBB dysfunction.

Sex is a critical biological variable in determining stroke outcome (Bushnell et al., 2018; Liu et al., 2009). Consistent with prior preclinical studies, female mice exhibited milder HT compared with adult males after tMCAO (Ahnstedt et al., 2020; Bushnell et al., 2018; Liu et al., 2009). In female mice, Ogerin reduced post-stroke edema at both proestrus/estrus and diestrus/metestrus stages. There was a trend of reduced HT at 24 h in Ogerin-treated females. The effect was not statistically significant, likely due to lower baseline hemorrhagic severity and limited N number. Nevertheless, the result suggests that Ogerin has similar effects on post-stroke HT and edema in both sexes.

In summary, the data presented here suggest that pharmacological potentiation of GPR68 with Ogerin likely protects BBB and reduces brain edema, at both ~24 and ~ 72 h after transient ischemia. The reduced brain infarct at 72 h suggests that Ogerin treatment can offer longer term protection in brain ischemia. Although the exact mechanism warrants further study, the current finding is provocative in that it suggests the potential cerebrovascular protective role of GPR68 activation. Together with previous studies on neuronal injury (Sun et al., 2024), our study suggests that Ogerin or similar GPR68 modulators are promising therapeutic reagents for neuroprotection following ischemic stroke.

## Data Availability

Data will be made available on request.

## References

[R1] Ahnstedt H, Patrizz A, Chauhan A, Roy-O’Reilly M, Furr JW, Spychala MS, D’Aigle J, Blixt FW, Zhu L, Bravo Alegria J, McCullough LD, 2020. Sex differences in T cell immune responses, gut permeability and outcome after ischemic stroke in aged mice. Brain Behav. Immun 87, 556–567. 32058038 10.1016/j.bbi.2020.02.001 PMC7590503

[R2] Bangdiwala SI, Bhargava A, O’Connor DP, Robinson TN, Michie S, Murray DM, Stevens J, Belle SH, Templin TN, Pratt CA, 2016. Statistical methodologies to pool across multiple intervention studies. Transl. Behav. Med 6, 228–235. 27356993 10.1007/s13142-016-0386-8 PMC4927450

[R3] Banzrai C, Bosookhuu O, Yadamsuren E, Dambasuren B, Turbat S, Erdenedalai T, Myadagsuren M, Munkhtur U, Baatar K, Boldbayar P, Avirmed T, Badrakh B, Ouyang M, Chen X, Wang X, Anderson CS, 2023. Incidence and outcomes for stroke in Ulaanbaatar, Mongolia, during 2019–21: a prospective population-based study. Lancet Glob. Health 11, e942–e952. 37119831 10.1016/S2214-109X(23)00130-4

[R4] Battey TW, Karki M, Singhal AB, Wu O, Sadaghiani S, Campbell BC, Davis SM, Donnan GA, Sheth KN, Kimberly WT, 2014. Brain edema predicts outcome after nonlacunar ischemic stroke. Stroke 45, 3643–3648. 25336512 10.1161/STROKEAHA.114.006884 PMC4295905

[R5] Bushnell C, Howard VJ, Lisabeth L, Caso V, Gall S, Kleindorfer D, Chaturvedi S, Madsen TE, Demel SL, Lee SJ, Reeves M, 2018. Sex differences in the evaluation and treatment of acute ischaemic stroke. Lancet Neurol. 17, 641–650. 29914709 10.1016/S1474-4422(18)30201-1

[R6] Bustamante A, Garcia-Berrocoso T, Rodriguez N, Llombart V, Ribo M, Molina C, Montaner J, 2016. Ischemic stroke outcome: a review of the influence of post-stroke complications within the different scenarios of stroke care. Eur. J. Intern. Med 29, 9–21. 26723523 10.1016/j.ejim.2015.11.030

[R7] Chen CH, Shoamanesh A, Colorado P, Saad F, Lemmens R, De Marchis GM, Caso V, Xu L, Heenan L, Masjuan J, Christensen H, Connolly SJ, Khatri P, Mundl H, Hart RG, Smith EE, 2024. Hemorrhagic transformation in noncardioembolic acute ischemic stroke: MRI analysis from PACIFIC-STROKE. Stroke 55, 1477–1488. 38690666 10.1161/STROKEAHA.123.045204

[R8] Farkas E, Rose CR, 2025. A dangerous liaison: spreading depolarization and tissue acidification in cerebral ischemia. J. Cereb. Blood Flow Metab 45, 201–218. 39535276 10.1177/0271678X241289756 PMC12000947

[R9] Flynn RW, MacWalter RS, Doney AS, 2008. The cost of cerebral ischaemia. Neuropharmacology 55, 250–256. 18573263 10.1016/j.neuropharm.2008.05.031

[R10] Garcia-Yebenes I, Sobrado M, Zarruk JG, Castellanos M, Perez de la Ossa N, Davalos A, Serena J, Lizasoain I, Moro MA, 2011. A mouse model of hemorrhagic transformation by delayed tissue plasminogen activator administration after in situ thromboembolic stroke. Stroke 42, 196–203. 21106952 10.1161/STROKEAHA.110.600452

[R11] Garcia-Yebenes I, Garcia-Culebras A, Pena-Martinez C, Fernandez-Lopez D, Diaz-Guzman J, Negredo P, Avendano C, Castellanos M, Gasull T, Davalos A, Moro MA, Lizasoain I, 2018. Iron overload exacerbates the risk of Hemorrhagic transformation after tPA (tissue-type plasminogen activator) Administration in Thromboembolic Stroke Mice. Stroke 49, 2163–2172. 30018160 10.1161/STROKEAHA.118.021540

[R12] Gerriets T, Walberer M, Ritschel N, Tschernatsch M, Mueller C, Bachmann G, Schoenburg M, Kaps M, Nedelmann M, 2009. Edema formation in the hyperacute phase of ischemic stroke. Laboratory investigation. J. Neurosurg 111, 1036–1042. 19408985 10.3171/2009.3.JNS081040

[R13] Gotoh O, Asano T, Koide T, Takakura K, 1985. Ischemic brain edema following occlusion of the middle cerebral artery in the rat. I: the time courses of the brain water, sodium and potassium contents and blood-brain barrier permeability to 125I-albumin. Stroke 16, 101–109. 3966252 10.1161/01.str.16.1.101

[R14] Hamann GF, Okada Y, del Zoppo GJ, 1996. Hemorrhagic transformation and microvascular integrity during focal cerebral ischemia/reperfusion. J. Cereb. Blood Flow Metab 16, 1373–1378. 8898714 10.1097/00004647-199611000-00036

[R15] He J, Fu F, Zhang W, Zhan Z, Cheng Z, 2022. Prognostic significance of the clinical and radiological haemorrhagic transformation subtypes in acute ischaemic stroke: a systematic review and meta-analysis. Eur. J. Neurol 29, 3449–3459. 35789517 10.1111/ene.15482

[R16] Hong JM, Kim DS, Kim M, 2021. Hemorrhagic transformation after ischemic stroke: mechanisms and management. Front. Neurol 12, 703258. 34917010 10.3389/fneur.2021.703258 PMC8669478

[R17] Huang FP, Zhou LF, Yang GY, 1998. The effect of extending mild hypothermia on focal cerebral ischemia and reperfusion in the rat. Neurol. Res 20, 57–62. 9471104 10.1080/01616412.1998.11740485

[R18] Huang XP, Karpiak J, Kroeze WK, Zhu H, Chen X, Moy SS, Saddoris KA, Nikolova VD, Farrell MS, Wang S, Mangano TJ, Deshpande DA, Jiang A, Penn RB, Jin J, Koller BH, Kenakin T, Shoichet BK, Roth BL, 2015. Allosteric ligands for the pharmacologically dark receptors GPR68 and GPR65. Nature 527, 477–483. 26550826 10.1038/nature15699 PMC4796946

[R19] Jiang X, Andjelkovic AV, Zhu L, Yang T, Bennett MVL, Chen J, Keep RF, Shi Y, 2018. Blood-brain barrier dysfunction and recovery after ischemic stroke. Prog. Neurobiol 163–164, 144–171. 10.1016/j.pneurobio.2017.10.001 PMC5886838 28987927

[R20] Jickling GC, Liu D, Stamova B, Ander BP, Zhan X, Lu A, Sharp FR, 2014. Hemorrhagic transformation after ischemic stroke in animals and humans. J. Cereb. Blood Flow Metab 34, 185–199. 24281743 10.1038/jcbfm.2013.203 PMC3915212

[R21] Jin G, Sun PZ, Singhal AB, Ayata C, Lo EH, 2010. First-order mathematical modeling of brain swelling in focal cerebral ischemia. Transl. Stroke Res 1, 65–70. 24323451 10.1007/s12975-009-0009-5

[R22] Khatri R, McKinney AM, Swenson B, Janardhan V, 2012. Blood-brain barrier, reperfusion injury, and hemorrhagic transformation in acute ischemic stroke. Neurology 79, S52–S57. 23008413 10.1212/WNL.0b013e3182697e70

[R23] Krueger M, Bechmann I, Immig K, Reichenbach A, Hartig W, Michalski D, 2015. Blood-brain barrier breakdown involves four distinct stages of vascular damage in various models of experimental focal cerebral ischemia. J. Cereb. Blood Flow Metab 35, 292–303. 25425076 10.1038/jcbfm.2014.199 PMC4426746

[R24] Krueger M, Hartig W, Frydrychowicz C, Mueller WC, Reichenbach A, Bechmann I, Michalski D, 2017. Stroke-induced blood-brain barrier breakdown along the vascular tree - no preferential affection of arteries in different animal models and in humans. J. Cereb. Blood Flow Metab 37, 2539–2554. 27683449 10.1177/0271678X16670922 PMC5531350

[R25] Kuts R, Frank D, Gruenbaum BF, Grinshpun J, Melamed I, Knyazer B, Tarabrin O, Zvenigorodsky V, Shelef I, Zlotnik A, Boyko M, 2019. A novel method for assessing cerebral Edema, infarcted zone and blood-brain barrier breakdown in a single post-stroke rodent brain. Front. Neurosci 13, 1105. 31680838 10.3389/fnins.2019.01105 PMC6805703

[R26] Li X, Xia K, Zhong C, Chen X, Yang F, Chen L, You J, 2024. Neuroprotective effects of GPR68 against cerebral ischemia-reperfusion injury via the NF-kappaB/Hif-1alpha pathway. Brain Res. Bull 216, 111050. 39147243 10.1016/j.brainresbull.2024.111050

[R27] Lin TN, He YY, Wu G, Khan M, Hsu CY, 1993. Effect of brain edema on infarct volume in a focal cerebral ischemia model in rats. Stroke 24, 117–121. 8418534 10.1161/01.str.24.1.117

[R28] Liu F, Yuan R, Benashski SE, McCullough LD, 2009. Changes in experimental stroke outcome across the life span. J. Cereb. Blood Flow Metab 29, 792–802. 19223913 10.1038/jcbfm.2009.5 PMC2748430

[R29] Longa EZ, Weinstein PR, Carlson S, Cummins R, 1989. Reversible middle cerebral artery occlusion without craniectomy in rats. Stroke 20, 84–91. 2643202 10.1161/01.str.20.1.84

[R30] Loubinoux I, Volk A, Borredon J, Guirimand S, Tiffon B, Seylaz J, Meric P, 1997. Spreading of vasogenic edema and cytotoxic edema assessed by quantitative diffusion and T2 magnetic resonance imaging. Stroke 28, 419–426 discussion 426–417. 9040700 10.1161/01.str.28.2.419

[R31] Paciaroni M, Agnelli G, Corea F, Ageno W, Alberti A, Lanari A, Caso V, Micheli S, Bertolani L, Venti M, Palmerini F, Biagini S, Comi G, Previdi P, Silvestrelli G, 2008. Early hemorrhagic transformation of brain infarction: rate, predictive factors, and influence on clinical outcome: results of a prospective multicenter study. Stroke 39, 2249–2256. 18535273 10.1161/STROKEAHA.107.510321

[R32] Ren H, Liu Y, Zhao M, Shen H, Nie S, Gao X, Huang Y, 2025. Stroke: epidemiology, risk factors, signaling pathways, and clinical management. MedComm (2020) 6, e70558. 41427019 10.1002/mco2.70558 PMC12711381

[R33] Rosenberg GA, Yang Y, 2007. Vasogenic edema due to tight junction disruption by matrix metalloproteinases in cerebral ischemia. Neurosurg. Focus 22, E4. 10.3171/foc.2007.22.5.5 17613235

[R34] Saria A, Lundberg JM, 1983. Evans blue fluorescence: quantitative and morphological evaluation of vascular permeability in animal tissues. J. Neurosci. Methods 8, 41–49. 6876872 10.1016/0165-0270(83)90050-x

[R35] Simard JM, Wilhelmy B, Tsymbalyuk N, Shim B, Stokum JA, Evans M, Gaur A, Tosun C, Keledjian K, Ciryam P, Serra R, Gerzanich V, 2024. Brain swelling versus infarct size: a problematizing review. Brain Sci. 14. 10.3390/brainsci14030229 PMC10968884 38539619

[R36] Spronk E, Sykes G, Falcione S, Munsterman D, Joy T, Kamtchum-Tatuene J, Jickling GC, 2021. Hemorrhagic transformation in ischemic stroke and the role of inflammation. Front. Neurol 12, 661955. 34054705 10.3389/fneur.2021.661955 PMC8160112

[R37] Sun W, Tiwari V, Davis G, Zhou G, Jonchhe S, Zha X, 2024. Time-dependent potentiation of the PERK branch of UPR by GPR68 offers protection in brain ischemia. Stroke 55, 2510–2521. 39224971 10.1161/STROKEAHA.124.048163 PMC11419283

[R38] Wang T, He M, Zha XM, 2020a. Time-dependent progression of hemorrhagic transformation after transient ischemia and its association with GPR68-dependent protection. Brain Hemorrhages 1, 185–191. 33575546 10.1016/j.hest.2020.10.001 PMC7872135

[R39] Wang T, Zhou G, He M, Xu Y, Rusyniak WG, Xu Y, Ji Y, Simon RP, Xiong ZG, Zha XM, 2020b. GPR68 is a neuroprotective proton receptor in brain ischemia. Stroke 51, 3690–3700. 33059544 10.1161/STROKEAHA.120.031479 PMC7678672

[R40] Xu J, Mathur J, Vessieres E, Hammack S, Nonomura K, Favre J, Grimaud L, Petrus M, Francisco A, Li J, Lee V, Xiang FL, Mainquist JK, Cahalan SM, Orth AP, Walker JR, Ma S, Lukacs V, Bordone L, Bandell M, Laffitte B, Xu Y, Chien S, Henrion D, Patapoutian A, 2018. GPR68 senses flow and is essential for vascular physiology. Cell 173, 762–775 e716. 29677517 10.1016/j.cell.2018.03.076 PMC5951615

[R41] Xu Y, Lin MT, Zha XM, 2020. GPR68 deletion impairs hippocampal long-term potentiation and passive avoidance behavior. Mol. Brain 13, 132. 32993733 10.1186/s13041-020-00672-8 PMC7526169

[R42] Yang GY, Betz AL, 1994. Reperfusion-induced injury to the blood-brain barrier after middle cerebral artery occlusion in rats. Stroke 25, 1658–1664.; discussion 1664–1655. 8042219 10.1161/01.str.25.8.1658

[R43] Yang C, Hawkins KE, Dore S, Candelario-Jalil E, 2019. Neuroinflammatory mechanisms of blood-brain barrier damage in ischemic stroke. Am. J. Physiol. Cell Physiol 316, C135–C153. 30379577 10.1152/ajpcell.00136.2018 PMC6397344

[R44] Yao Y, 2019. Basement membrane and stroke. J. Cereb. Blood Flow Metab 39, 3–19. 30226080 10.1177/0271678X18801467 PMC6311666

[R45] Zha XM, Xiong ZG, Simon RP, 2022. pH and proton-sensitive receptors in brain ischemia. J. Cereb. Blood Flow Metab 42, 1349–1363. 35301897 10.1177/0271678X221089074 PMC9274858

[R46] Zhou G, Wang T, Zha XM, 2021. RNA-Seq analysis of knocking out the neuroprotective proton-sensitive GPR68 on basal and acute ischemia-induced transcriptome changes and signaling in mouse brain. FASEB J. 35, e21461. 33724568 10.1096/fj.202002511R PMC7970445

